# Channeling is a distinct class of dissolution in complex porous media

**DOI:** 10.1038/s41598-023-37725-6

**Published:** 2023-07-13

**Authors:** Hannah P. Menke, Julien Maes, Sebastian Geiger

**Affiliations:** 1 Institute of GeoEnergy Engineering, Heriot-Watt University, Edinburgh, UK; 2 Department of Geoscience and Engineering, Delft University of Technology, Delft, The Netherlands

**Keywords:** Hydrology, Carbon capture and storage, Geothermal energy

## Abstract

The traditional model of solid dissolution in porous media consists of three dissolution regimes (uniform, compact, wormhole)—or patterns—that are established depending on the relative dominance of reaction rate, flow, and diffusion. In this work, we investigate the evolution of pore structure using numerical simulations during acid injection on two models of increasing complexity. We investigate the boundaries between dissolution regimes and characterize the existence of a fourth dissolution regime called channeling, where initially fast flow pathways are preferentially widened by dissolution. Channeling occurs in cases where the distribution in pore throat size results in orders of magnitude differences in flow rate for different flow pathways. This focusing of dissolution along only dominant flow paths induces an immediate, large change in permeability with a comparatively small change in porosity, resulting in a porosity–permeability relationship unlike any that has been previously seen. This work suggests that the traditional conceptual model of dissolution regimes must be updated to incorporate the channeling regime for reliable forecasting of dissolution in applications like geothermal energy production and geologic carbon storage.

## Introduction

The traditional conceptual model of mineral dissolution in porous media consists of three ‘dissolution regimes’ that guide prediction of flow and transport during reactive dissolution^1–4^. Accurate identification of these regimes is essential as it is the regime that ultimately determines the evolution of permeability. Moving between regimes can result in orders of magnitude increase in permeability change with porosity evolution. As such, accurate prediction of mineral dissolution in porous media is crucial for a wide range of subsurface applications, including CO$_2$ sequestration and geothermal power generation^5, 6^ where failure to predict the changes in permeability can lead to poor fluid injection efficiency and potentially irreversible reservoir damage^7, 8^.

**Figure 1:**
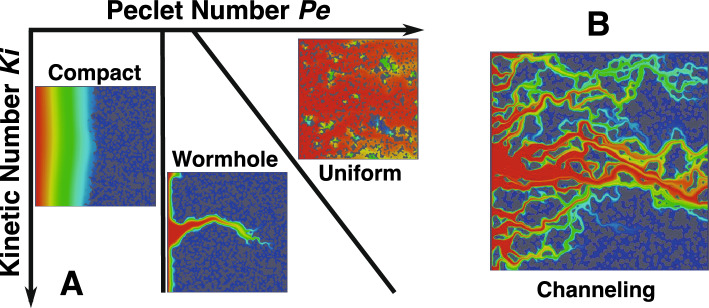
(**A**) Schematic depiction of dissolution regimes in the Péclet number—kinetic number space. (**B**) This paper modifies this traditional conceptual model by adding the channeling regime.

The balance between flow, diffusion, and reaction rates determines which dissolution pattern develops during reactive flow in a porous medium^9^. When flow is slow compared to reaction rate, the face of the porous medium closest to the inlet will dissolve and result in compact dissolution. When flow is fast compared to the reaction rate, acidic fluid is quickly distributed throughout the pore spaces and the medium dissolves uniformly. At intermediate flow rates, the acidic fluid etches a wide pathway through the porous medium in the direction of flow and forms a wormhole. These regimes can be predicted based on the Péclet number *Pe* (the ratio of advective to diffusive transport) and the Kinetic number *Ki* (the ratio of chemical reaction to diffusive transport). However, these dissolution regimes do not take into account the structural heterogeneity of complex porous media, because they were first identified (Fig. 1) before the technology was developed to observe or model reactive flow at the scale of grains and pores. Thus, they are problematic when quantifying the relationships between flow, reaction, and pore structure.

Recent advances in X-ray-CT imaging techniques^10, 11^ have enabled direct observation and quantification of dissolution-induced changes in the pore structure and provided insight into influences of structural heterogeneity, flow, and reaction rate on dissolution regime. Several experimental studies have observed mineral dissolution at the pore-scale in reservoir rock samples^12–16^. Others^17–20^ studied the dissolution dynamics in situ during fast flow in rocks of varying complexity, observing uniform dissolution in a structurally simple rock, but the opening of preferential flow pathways in the more complex rock samples. This path-widening did not progress longitudinally with flow, as is the case for wormholes, but instead opened everywhere along the dominant flow channel and was thus named ‘channeling’. This regime was later confirmed^21^ by observations of channeling in both fractured and vuggy rock samples. However, as of yet no in-depth experimental characterisation of the conditions required for channeling has been performed, and thus no new conceptual model has been proposed that includes channeling.

Pore-scale experimental techniques are often complemented by advances in numerical simulations that give insight into the complex relationship between pore structure, flow, and reaction. However, limitations in the numerical methods have not allowed for flow to be simulated at the high flow rates seen near reservoir injection wells^22–24^, which limits the range of dissolution regimes that can be studied. Several studies^25, 26^ have attempted a comprehensive numerical investigation of the full spectrum of pore-scale dissolution regimes (Fig. 1), but these were restricted to relatively homogeneous domains with minor differences in pore structure between models and small differences in flow rate. Channeling has thus not been characterised in numerical models at the pore-scale by any study to date because either the numerical capabilities for high flow rates or structural complexity in the model were lacking. Therefore, the placement of the boundaries between wormhole, channeling, and uniform dissolution regimes are unknown and the conceptual model of dissolution is missing information vital for accurate modelling of dissolution.

**Figure 2:**
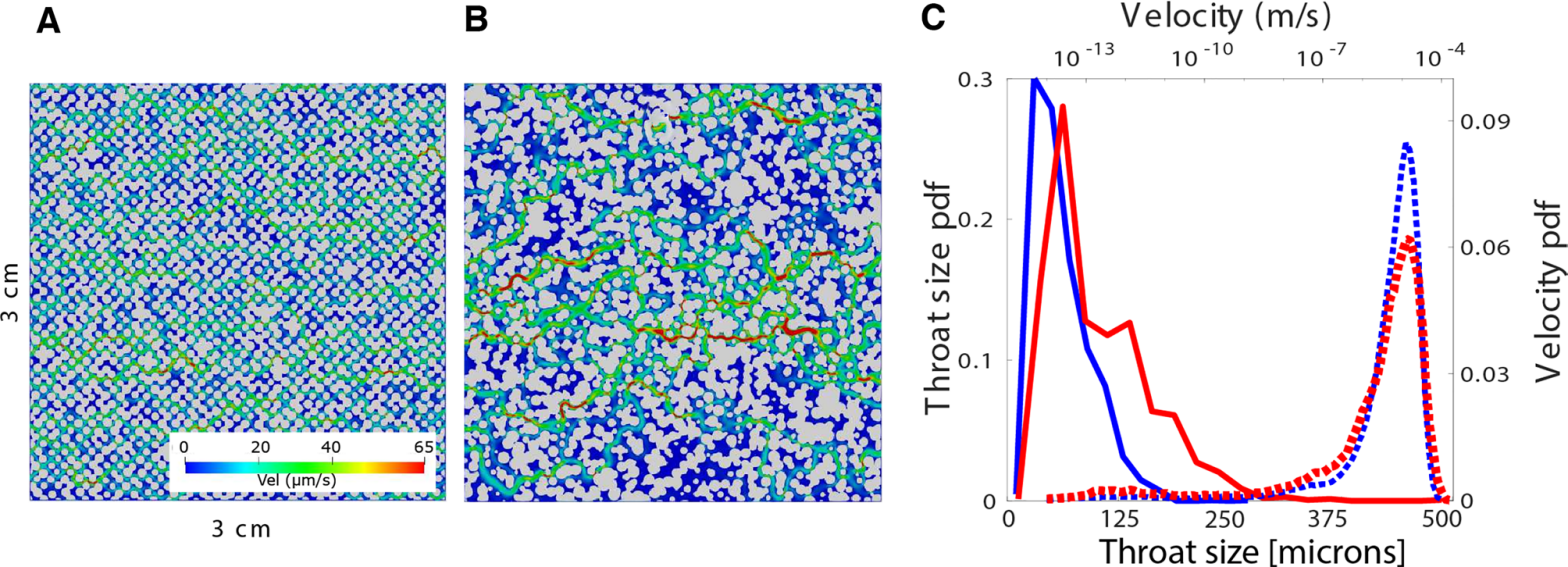
(**A**) Model A. (**B**) Model B. The grains (gray) are rendered with the velocity field of the pore space (color) computed using an injection rate of 0.4 mL/min and a resolution of 2.5 $\upmu$m per pixel. (**C**) Results showing the histogram of throat size (solid) and velocity (dotted) for Model A (blue) and Model B (red). The characteristic length *L* is $1.125 \times 10^{-4}$ m for Model A and $1.251 \times 10^{-4}$ m for Model B.

The work presented here is a numerical investigation into how pore-space complexity changes the conceptual model of dissolution regimes and how the channeling regime fits into our broader understanding of dissolution. Two synthetic 2D pore structures with varying levels of heterogeneity were created stochastically and their structural complexity characterized (Fig. 2). A series of 26 numerical simulations was performed on each of the geometries by injecting acid at different flow and reactive conditions using our new highly efficient open source numerical solver GeoChemFoam^27–30^, which is based on the Open Source Computational Fluid Dynamics toolbox OpenFOAM^31^ and has been previously benchmarked against experimental datasets from^17, 26^. We observe that many of the model scenario results do not fit the conceptual model of the three traditional dissolution regimes and have fundamentally categorically distinct porosity–permeability relationships. We show that these four dissolution regimes can be distinguished using the moments (mean, standard deviation, skewness, and kurtosis) of the distributions of pore throat size and acid concentration. We then employ hierarchical agglomerative clustering^32^ to provide a quantitative measure of identifying the channeling regime and differentiating channeling from the other three regimes. Finally, we provide an updated conceptual model of dissolution regimes that includes channeling.

## Results

### Numerical observations of pore-scale dissolution

The models are constructed with beads of a non-uniform spacing in order to allow for preferential flow paths and reaction infiltration instabilities. First, a relatively homogeneous geometry was created with a small random deviation in both grain radius and placement of the grains (Model A, Fig. 2A). Model A represents the smallest random deviation that resulted in the inducement of flow instabilities. Structural complexity was then increased by adding a larger random deviation of both grain radius and placement to create an increasingly heterogeneous geometry (Model B, Fig. 2B). The distributions of throat sizes and velocity of Model A and Model B are presented in Fig. 2C. Model A has velocity and pore throat size distributions that are narrow, while Model B shows a wide tail representing the focusing of flow into the preferential flow paths through larger pore throats. Additional details on geometry creation and the numerical modelling are included in Supplementary material.

For each geometry, we perform 26 simulations to identify the boundaries between dissolution regimes. The model solves the quasi-steady state Navier–Stokes equations and advection–diffusion of reactant in the pore space using a finite-volume discretization on an unstructured hybrid mesh consisting of hexahedral and split-hexahedral elements^31^. The numerical model, including meshing, time-stepping and convergence, is presented in detail in Supplementary material. A simplified chemical model is employed representing dissolution of calcite mineral during acid injection, with one fluid component and one reaction component^22, 26, 30^. The molecular diffusion is the constant $D=10^{-9}$ m$^2$/s. The displacement of the fluid–solid interface is handled using the Arbitrary Eulerian Lagrangian (ALE) method. Acid is injected from the left boundary at constant concentration and flow rate and the simulations are ended either when the porosity increases to 1.6 times the initial porosity or the permeability reaches a value 100 times larger than the initial permeability.

The relative importance of advection and reaction rate to molecular diffusion is characterized by the Péclet number $Pe=UL/D$ and Kinetic number $Ki=kL/D$, (also known as Da(II)) where *U* (m/s) is the average pore velocity, *L* (m) is the average width of the flow pathways and *k* (m/s) is the reaction constant. The first Daköhler number Da(I) is defined as the ratio of the Kinetic and Péclet numbers and describes the relative importance of advection and reaction rate. Details on how to calculate *Pe*, *Ki*, *U* and *L* are presented in Supplementary material. For each simulation, the flow rate and reaction constant are adjusted to obtain the desired *Pe* and *Ki* at time=0.

**Figure 3:**
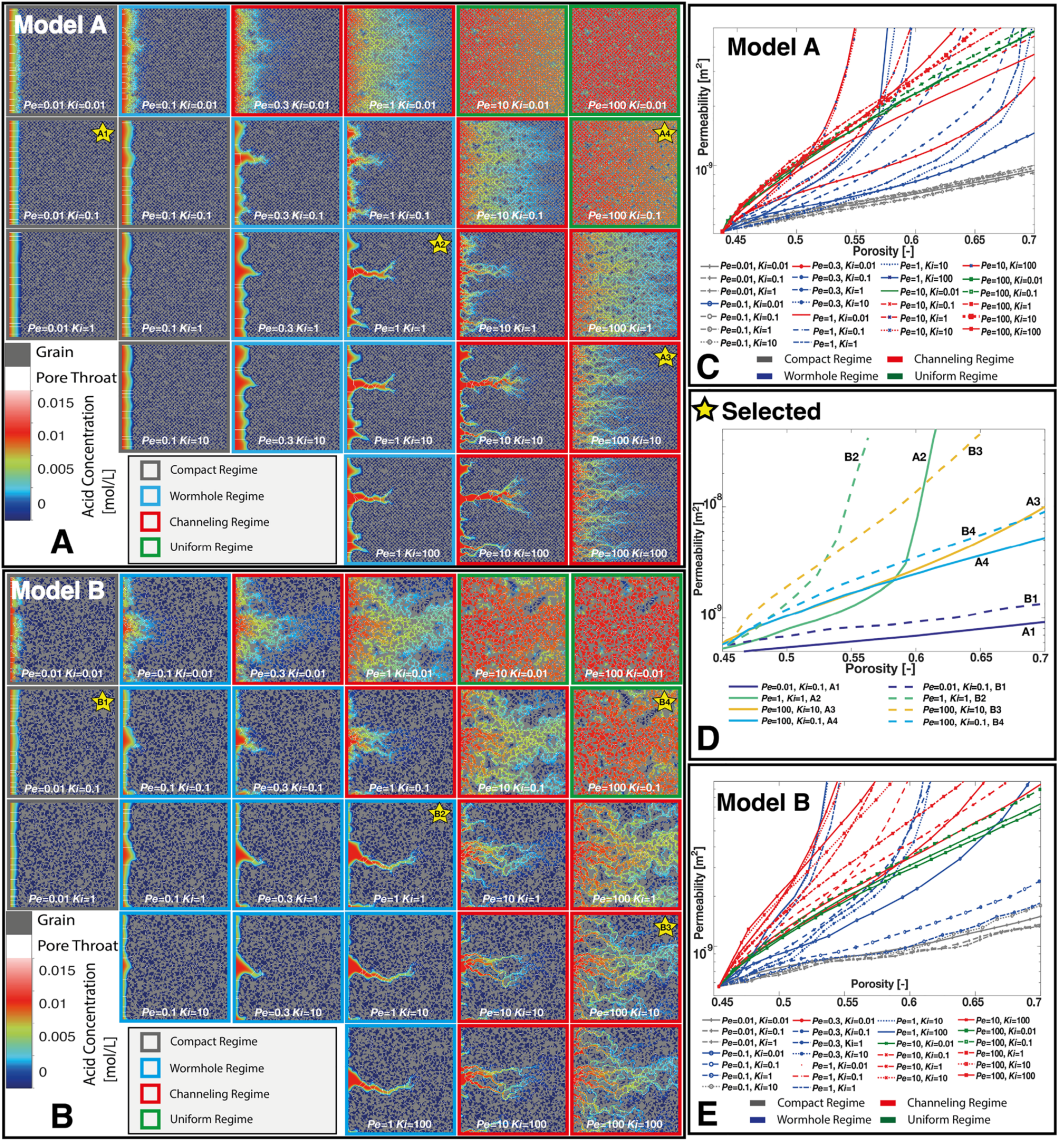
Pore structure and acid concentration during mineral dissolution in (**A**) Model A and (**B**) Model B at *Pe* and *Ki* ranging from 0.01 to 100 at a porosity of 0.57. The solid phase is rendered in grey and the acid concentration in colors. The pore throats extracted using a watershed algorithm are shown in white. Simulations categorised in the compact, wormhole, and uniform regimes are outlined in gray, blue, and green, respectively, while simulations that do not fit into any traditional regime are outlined in red and designated channeling. (**C**,**E**) Porosity–permeability curves for all the Model A and Model B simulations, respectively. (**D**) Porosity–permeability curves for selected (starred) simulations.

Maps showing the distribution of the injected acid concentration at the time where dissolution has increased the porosity from  0.45 to  0.5 are presented (Fig. 3A and B). Videos of the dynamic evolution of dissolution are provided in Supplementary material. In Fig. 3A, we observe the three traditional regimes for Model A: compact dissolution (gray), wormhole (blue) and uniform dissolution (green). The cases at the boundary between wormhole and uniform dissolution, outlined in red, are traditionally classified as (ramified) wormholes^25, 26^. However, here we observe they exhibit characteristics that contradict the wormholing concept. Rather than one ramified wormhole that has very little change in permeability until breakthrough (e.g. $Pe=1, Ki=0.1$), these include a very large number of small dissolution channels that extend towards the outlet of the model, resulting in a porosity–permeability evolution with similar curvature to those of uniform dissolution, but with a larger change in permeability with porosity as dissolution is present in these pathways at the outlet almost instantaneously. In these cases, there is a direct correspondence between dissolution pathways and initial fast flow paths (Fig. 2A). The most dominant flow paths are dissolved first, which leads to an initial increase in permeability that is higher than that observed for uniform dissolution (e.g. $Pe=100, Ki=10$) (Fig. 3A). We will demonstrate that this regime is channeling, as identified in previous experimental studies^18, 21^.

**Figure 4:**
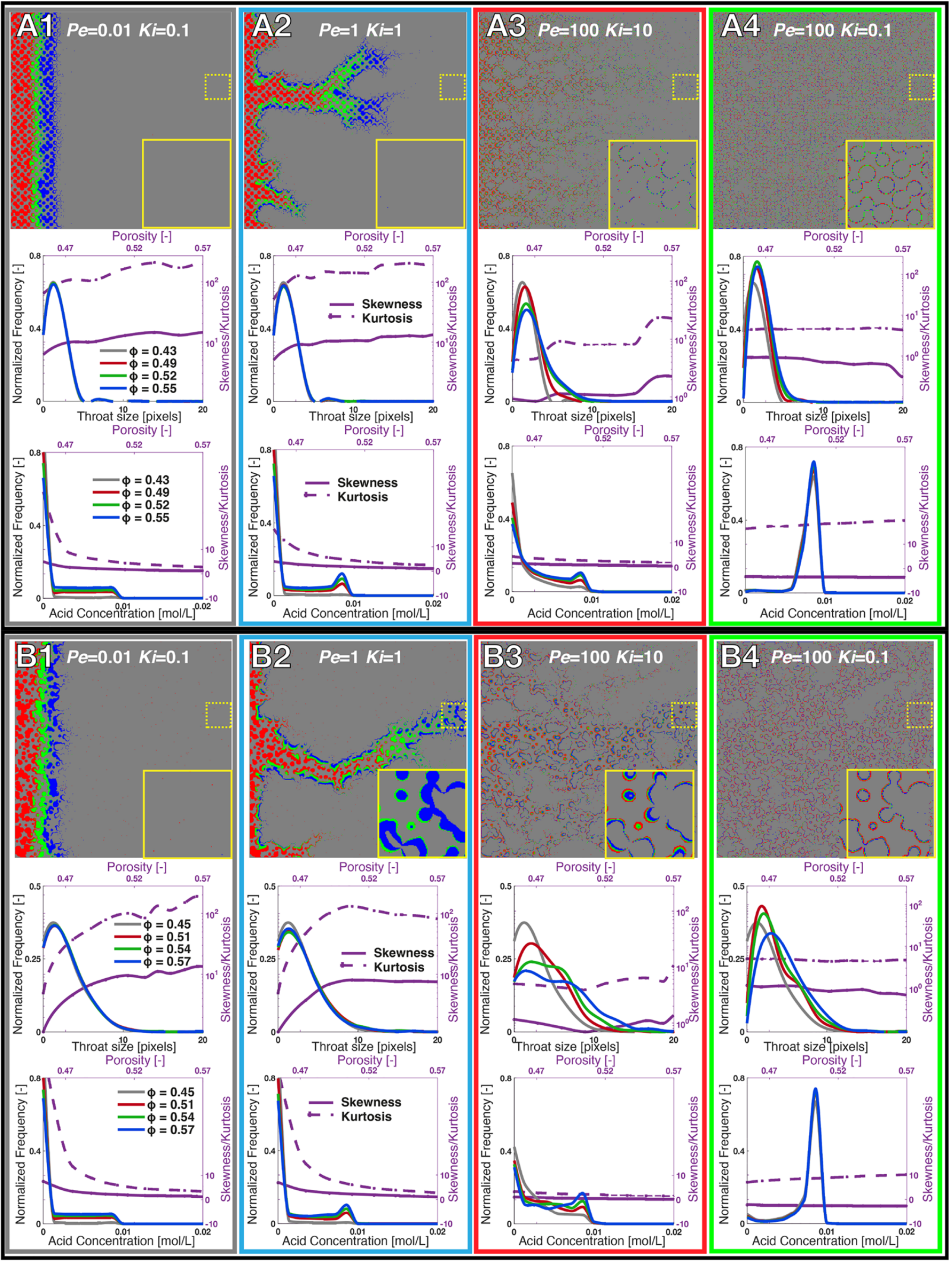
(Rows 1 and 4) Dissolution with change in porosity as a proxy for time for select simulations of Model A (A1–4) and B (B1–4). In the top of each example, the dissolved pore space is shown at different porosity values with undissolved being grey, and red, green, and blue showing dissolution at subsequent times. The solid yellow squares are magnified regions of interest of the outlet of the model during dissolution which are outlined as dashed yellow squares. (Rows 2 and 4) The pore throat size distributions of Model A (row 2) and Model B (row 4) of the above simulation at the porosity values depicted in rows 1 and 4 (grey, red, green, blue). The skewness (solid line) and kurtosis (dashed line) of pore throat size are shown with increasing porosity on the right axes (purple). (Rows 3 and 6) The concentration distributions of Model A (row 3) and Model B (row 4) at the same porosity values depicted in rows 1 and 4 (grey, red, green, blue). The skewness (solid line) and kurtosis (dashed line) of the concentration distributions are shown with increasing porosity using the right axes (purple).

The existence of channeling becomes more apparent as structural complexity increases in Model B (Fig. 3B), where we again observe a number of cases (outlined in red) that cannot be classified using any of the three traditional regimes and instead follow the same convex porosity–permeability (Fig. 4E) trends as those in Model A (Fig. 4C). In addition, the increased structural complexity has increased the order of the porosity–permeability change with faster widening of the channels in the more heterogeneous cases. In all of the channeling cases, the permeability increases faster and attains a larger value than for uniform dissolution and is faster than for the more structurally homogeneous cases in Fig. 3A.

To illustrate the impact of pore space heterogeneity on dissolution regime, the evolution of the dissolution patterns and the throat size and concentration distributions for selected cases of Model A and B are shown (Fig. 4D). The details of image analysis techniques used to extract these metrics can be found in Supplementary material. The corresponding evolutions of the porosity–permeability relationships are shown in Fig. 3D. In the compact dissolution cases (A1, B1), the dissolution is transport-limited and creates large throats at the front of the model that result in a large skewness and kurtosis in throat size. Conversely, as the dissolution front advances, the highly concentrated acid spread into more of the pore space and the skewness and kurtosis of the concentration distributions decrease. The small deviations in the dissolution front in Model B result in a larger overall skewness and kurtosis of throat size and concentration and a larger slope in the porosity–permeability relationship than Model A. However, even at the largest porosity shown for Model B (porosity = 0.57), the dissolution front remains stable, and there is no dissolution near the outlet, so the overall change in permeability is low. When we apply a power law fit to the porosity–permeability relationship, we find the relative small exponent of 1.5–2.

In the cases A2 and B2, the dissolution front becomes unstable, and advection and reaction compete as the dissolution etches pathways (wormholes) through the models. Large pore throats are created both at the fronts and inside the wormholes, which result in a large increase in the skewness and kurtosis of pore throat size and a widening of the pore throat size distribution through time. The concentration distributions develop a peak indicative of a preferential flow path through the model with corresponding decreases in skewness and kurtosis, as the wormhole carries the acid towards the outlet. In Model A, similar competition between flow paths results in a porosity–permeability relationship (Fig. 3D) that is similar to compact dissolution. There is a rapid increase in permeability once the wormhole is established in the fastest flow pathway, but has a much higher exponent of 11. The preferential flow path is more dominant in Model B. We observe less competition initially with breakthrough of the wormhole to the outlet occurring earlier with a larger increase in permeability and an exponent as high as 19.

In cases A3 and B3, the pore throats in the preferential flow pathways are dissolved across the entire domain at the very beginning of the simulations, which creates a fat tail in the throat size distributions and peaks in the concentration distributions. Notably, the skewness and kurtosis of concentration show very little change due to the broad spread of the acid even from the beginning of the simulations. Flow is focused in these channels and there is little dissolution in the slower flowing areas of the pore space. This focused dissolution results in a porosity–permeability relationship of power law exponent 6–12. In Model B, the structural complexity is higher, and there are fewer fast flowing channels, however, they are more important and result in a higher order porosity–permeability relationship. The dissolution converges towards these fast channels and the flow inside them becomes so dominant that no wormhole forms in the domain. For channeling, flow is stable and the dissolution channels are instantaneously established as the dominant flow pathways and then become wider as the porosity increases.

In cases A4 and B4 the dissolution is reaction-limited and uniform across the domains, with no preferential pathways forming in either Model A or B. The kurtosis and skewness of pore throat size across the domains is flat as all flow paths are widened together. The concentration distribution has a large peak at the injection concentration which increases only slightly throughout the simulations as more of the model dissolves. Here, the skewness of concentration is below 0, which is contrary to all other dissolution regimes. During uniform dissolution the increased structural heterogeneity results in only a small increase in the power law exponent of the porosity–permeability relationship from 5 to 6.

### Channeling: a new class of dissolution regime

**Figure 5:**
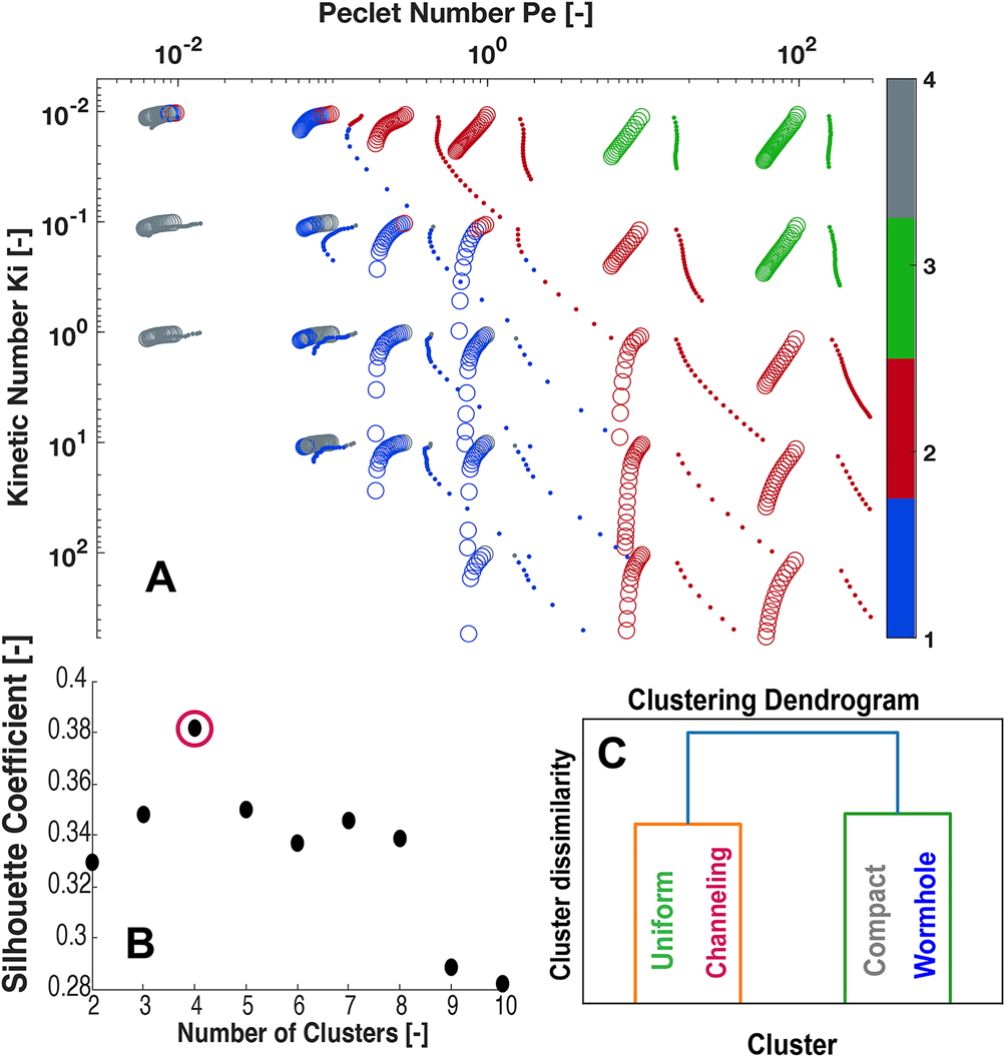
Hierarchical agglomerate clustering of dissolution using the mean, standard deviation, skewness, and kurtosis of concentration and pore throat size. (**A**) Each circle (Model A) and dot (Model B) represents a single time-step in one numerical experiment, and is color coded by cluster by the clustering algorithm, according to the dendrogram in (**C**). (**B**) Silhouette Coefficient of the number of clusters. (**C**) The dendrogram of the cluster labels.

We quantitatively identify dissolution regime by clustering the four moments (mean, standard deviate, skewness, and kurtosis) of the distributions in concentration and throat size at each time step (Fig. 5). We used hierarchical agglomerative clustering for a range of numbers of clusters from 2 to 10 shown in Fig. 5B. The Silhouette Coefficient (SC) is used to rank the optimal number of clusters where a higher index indicates that clusters are dense and well separated. For our group of simulations, the highest SC was observed with 4 clusters. This clustering (Fig. 5A) identifies channeling as independent regime and is in agreement with our visual characterization and physical understanding of the numerical experiments.

The clustering dendrogram (Fig. 5C) gives insight into how the clustering algorithm determines each cluster boundary. First the channeling/uniform regimes split from the wormhole/compact regimes, followed by the compact and wormhole regimes, and finally channeling and uniform regimes. The order of splitting indicates that the difference in dissolution behavior is greatest between the channelling/uniform regimes and the wormhole/compact regimes, and smallest between the channeling and uniform regimes, which confirms our assertion that channeling is distinct from wormhole formation. The clustering also indicates that some simulations straddle the boundary between regimes, beginning in one regime and ending in another as the dissolution changes the distribution of flow within the porous medium and flow becomes more or less stable in preferential flow pathways. This is consistent with our analysis of the dissolution progress shown in Figs. 3 and 4.

**Figure 6:**
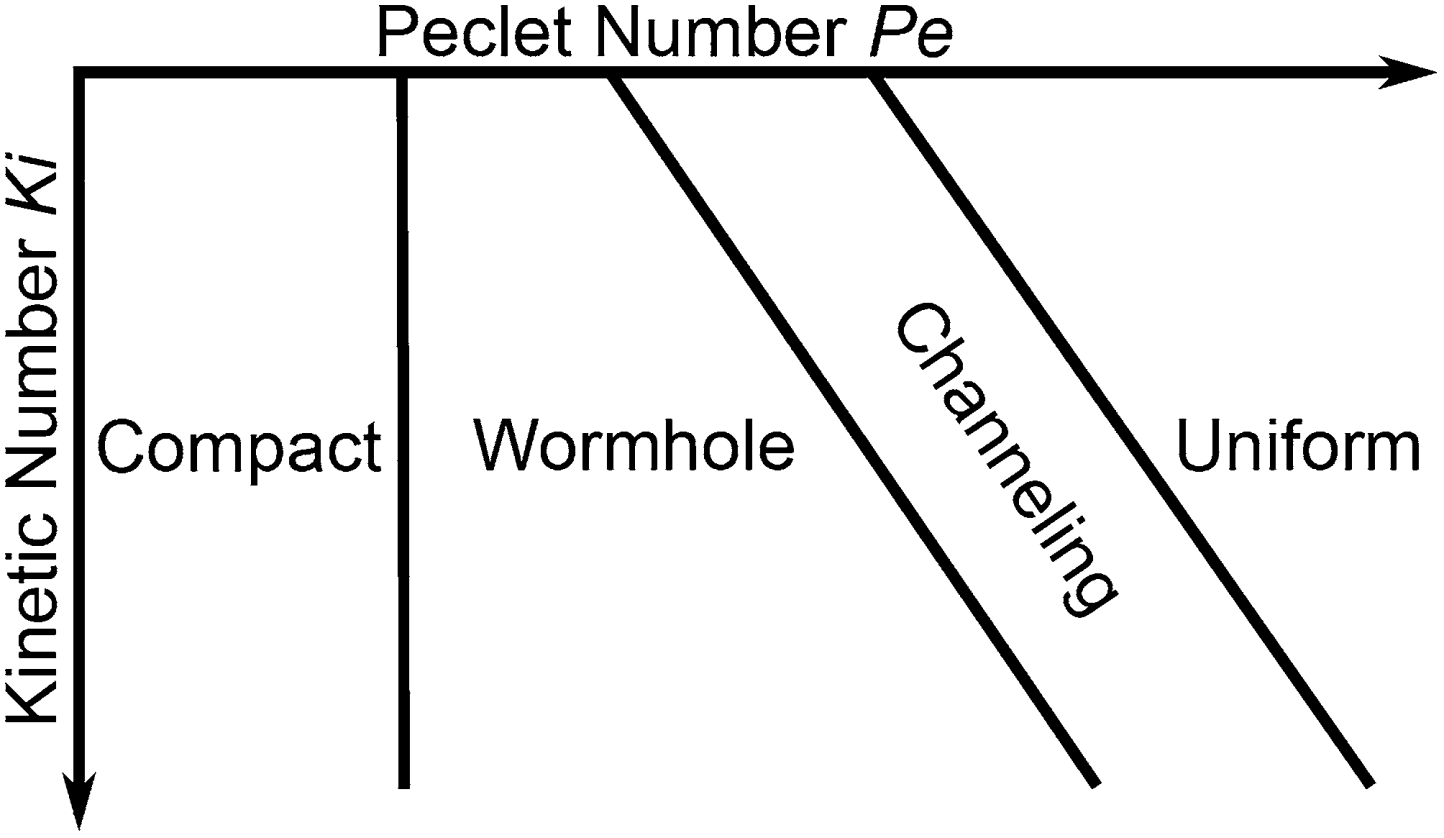
The Péclet number—kinetic number space with updated dissolution regimes. Channeling exists as a distinct regime between wormhole and uniform.

We present our updated conceptual model of dissolution regimes in Fig. 6. Channeling is a distinct regime between wormhole formation and uniform dissolution. In more heterogeneous structures, the relative importance of already existing flow paths increases, leading to the formation of wormholes and channels faster, with a higher order.

## Discussion: reconciling the pore scale with the continuum scale

We have characterised the dissolution regime of channeling, identified its location within the $Pe-Ki$ space, and quantified its relationship to wormhole formation and uniform dissolution. Previous experimental work in 3D has reported the porosity–permeability of channeling to have a power law order of between 7 and 11^33^ and the uniform regime to have an order of 5^17^, which is consistent with our 2D observations of power law order 6–12 for channeling and 5–6 for the uniform regime. This indicates that the 2D results are likely to be directly extendable to 3D.

Characterisation of dissolution regimes are crucial for providing accurate porosity–permeability relationships for Darcy and reservoir-scale models. In contrast to other pattern formations such as viscous fingering in multi-phase flow, both the location and the conditions under which dissolution follows pre-existing flow paths is important. Wormholes develop from the pore-scale as micron-scale ramifications that merge and expand to eventually form dissolution pathways that impact flow at the field-scale. Similarly channeling will influence flow during dissolution from the pore- to the field-scale provided that scale-dependent structural complexity exists, as for example with the presence of vugs, fractures and faults^21^. Predicting such development of dissolution patterns at the field-scale requires an accurate estimation of the evolving permeability of the dissolving matrix^34^.

This unique research provides a first-ever characterisation of the channeling regime. Channeling occurs in heterogeneous porous media, where differences in pore throat sizes cause dissolution to widen preferential flow pathways. This study is the first step towards understanding the multi-scale interactions between structure and dissolution in more complex multi-scale domains such as carbonate rocks where knowledge of how the pore space dissolves at the scale of grains and pores can be incorporated into field scale models. Indeed, in the carbonate reservoirs typically considered for industrial geologic carbon storage applications with a representative calcite reaction constant and carbonate reference pore throat sizes^19^, *Ki* will range between 0.1 and 100. Therefore at sufficiently fast flow rates, the dissolution will be in the channeling regime. Accurate characterisation of the channeling regime is thus vital for accurate prediction of dissolution during many commercial processes essential for the clean energy transition. This method and results clearly show that a complete understanding of the channeling regime will be essential for any implementation of the advection–diffusion–reaction equations across a broad range of applications including flow organisation during magma melt^35, 36^, diagenesis^37, 38^, and other geological processes^39^, drug delivery systems^40^, contaminant transport in underground reservoirs^41–43^, and virus spreading dynamics^44^.

## Materials and methods

All numerical simulations were performed using GeoChemFoam on Intel Xeon processors (24 cores). For each image, an unstructured mesh is created within the pore-space using OpenFOAM utility *snappyHexMesh*. For each time-step, velocity and acid concentration fields are solved. Then the reaction rate and the velocity of the dissolving faces are calculated and the mesh is updated. Mesh quality is checked at the end of each time-step and if the skewness is too large, the domain is completely remeshed. Since GeoChemFoam uses steady-state formulations of flow and transport, it can be applied with very large time-steps ($CFL\approx 1000$), allowing for large speed-ups in computation time. Details of the geometry creation, analysis, meshing, numerical method, and time stepping strategy are presented in^30^ and in Supplementary material. The original geometries and output files can be downloaded from our Zenodo dataset archive, the geometry creation scripts are on github and an example input deck is on the GeoChemFoam wiki.

### Governing equations

Under isothermal conditions and in the absence of gravitational effects, fluid motion in the pore-space is governed by the incompressible Navier–Stokes equations,

1 $$\begin{aligned}{} & {} \nabla \cdot {\textbf{u}} = 0, \end{aligned}$$

2 $$\begin{aligned}{} & {} \frac{\partial {\textbf{u}}}{\partial t}+ \nabla \cdot \left( {\textbf{u}}\otimes {\textbf{u}}\right) =-\nabla p +\nu \nabla ^2{\textbf{u}}, \end{aligned}$$

with the continuity condition at the fluid-solid interface $\Gamma$,

3 $$\begin{aligned} \rho \left( {\textbf{u}}-{\textbf{w}}_s\right) \cdot {\textbf{n}}_{s}=-\rho _s{\textbf{w}}_s\cdot {\textbf{n}}_s \quad \text {at}\,\, \Gamma , \end{aligned}$$

where ${\textbf{u}}$ (m/s) is the velocity, *p* (m$^2$/s$^2$) is the kinematic pressure, $\nu$ (m$^2$/s) is the kinematic viscosity, $\rho$ (kg/m$^3$) is the fluid density, $\rho _s$ (kg/m$^3$) is the solid density, $\mathbf {n_s}$ is the normal vector to the fluid–solid interface pointing toward the solid phase, and $\mathbf {w_s}$ (m/s) is the velocity of the fluid–solid interface, which is controlled by the surface reaction rate *R* (kmol/m$^2$/s) such that

4 $$\begin{aligned} {\textbf{w}}_s=\frac{M_{ws}}{\rho _s}R{\textbf{n}}_s, \end{aligned}$$

where $M_{ws}$ is the molecular weight of the solid. The concentration *c* (kmol/m$^3$) of a species in the system satisfies an advection–diffusion equation

5 $$\begin{aligned} \frac{\partial c}{\partial t}+ \nabla \cdot \left( c{\textbf{u}} \right) = \nabla \cdot \left( D\nabla c\right) , \end{aligned}$$

where *D* (m$^2$/s) is the diffusion coefficient. The chemical reaction occurs at the fluid–solid interface $\Gamma$, such that

6 $$\begin{aligned} \left( c\left( {\textbf{u}}-{\textbf{w}}_s\right) -D\nabla c \right) \cdot {\textbf{n}}_{s}=\zeta R \quad \text {at} \,\, \Gamma , \end{aligned}$$

where $\zeta$ is the stoichiometric coefficient of the species in the reaction. In this work, we assume that the surface reaction rate depends only on the concentration of one reactant species, following

7 $$\begin{aligned} R=k_cc, \end{aligned}$$

where $k_c$ (m/s) is the reaction constant. At the inlet, the boundary conditions are constant flow rate *Q* (m$^3$/s) and constant reactant concentration $c_i$ (kmol/m$^3$). To limit inlet boundary effect, the velocity is extrapolated from a zero gradient rather than taken as constant^31^. At the outlet, the boundary conditions are constant pressure $p_0$ (m$^2$/s) and a zero gradient for velocity and reactant concentration.

### Dimensionless analysis

The flow, transport and reaction conditions are characterized by the Reynolds number

8 $$\begin{aligned} Re=\frac{UL}{\nu }, \end{aligned}$$

which quantifies the relative importance of inertial to viscous forces, the Péclet number,

9 $$\begin{aligned} Pe=\frac{UL}{D}, \end{aligned}$$

which quantifies the relative importance of advective and diffusive transport, and the Kinetic number,

10 $$\begin{aligned} Ki=\frac{k_cL}{D}, \end{aligned}$$

which quantifies the relative importance of chemical reaction and diffusive transport. Here *U* and *L* are the reference pore-scale velocity and length. The Kinetic number characterized if the chemical reaction at the surface of solid grains is in the reaction-limited ($Ki<1$) or transport-limited ($Ki>1$) regime. The Damköhler number *Da*, which is the ratio of Kinetic and Péclet numbers, is also a relevant quantity. *Da* quantifies the relative importance of reaction to advective transport globally, but not locally as the reactant can only be transported to the solid surface by diffusion (Eqs. 3, 6). In this study, we assume that we are in the creeping flow regime ($Re<<1$) so that the dissolution regime is only dependent on *Pe* and *Ki*. In addition, the reactant strength, defined as

11 $$\begin{aligned} \beta =\frac{c_{i}M_{ws}}{\zeta \rho _s}, \end{aligned}$$

characterised how many kg of solid are dissolved by a kg of reactant. The pore-scale reference velocity is chosen as the average pore velocity, defined as

12 $$\begin{aligned} U=\frac{U_D}{\phi }, \end{aligned}$$

where $\phi$ is the porosity of the domain and $U_D$ (m/s) is the Darcy velocity, defined as

13 $$\begin{aligned} U_D=\frac{Q}{A}, \end{aligned}$$

where *A* (m$^2$) is the cross-sectional area of the domain. The pore-scale reference length scale *L* is defined as

14 $$\begin{aligned} L=\sqrt{\frac{12K}{\phi }}, \end{aligned}$$

where *K* (m$^2$) is the permeability of the domain, and the parameter 12 is a constant defined so that the pore-scale length scale corresponds to the tube size for a capillary bundle of constant size. The permeability can be calculated as

15 $$\begin{aligned} K=-\frac{\nu U_DL_D}{\Delta P}, \end{aligned}$$

where $L_D$ is the length of the domain and $\Delta P$ is the pressure drop between inlet and outlet. The pressure is a constant at the outlet, but not at the inlet (constant flow rate boundary condition). Therefore, the pressure drop is defined as^45^

16 $$\begin{aligned} \Delta P = -\frac{1}{Q}\frac{dW_P}{dt}, \end{aligned}$$

where $W_P$ is the work done by the pressure force in the domain. Eqs. (15) and (16) together denote that, for an equivalent flow rate, a higher permeability corresponds to a lower energy dissipation in the domain. The rate of energy dissipation $\frac{dW_P}{dt}$ can be calculated as

17 $$\begin{aligned} \frac{dW_P}{dt}=-\int _V{\nabla p\cdot udV}. \end{aligned}$$

### Quasi–static assumption

Dissolution of a solid grain is typically orders of magnitude slower than reactant transport. This is characterised in our numerical model by $\beta Da<<1$ and $\beta Ki<<1$. For example, for dissolution of calcite ($M_{ws}=100$ kg/kmol, $\rho _s=2710$ kg/m$^3$) by an acid at pH=2 ($c_i=0.01$ kmol/m$^3$), the reactant strength $\beta$ is equal to $3.69\times 10^{-4}$. Therefore, as long as $Pe<100$ and $Ki<100$, the displacement of the solid interface is slow compared to the transport of reactant in the domain, and flow (Eq. 2) and transport (Eq. 5) can be assumed to be in a quasi-static state

18 $$\begin{aligned} \nabla \cdot \left( {\textbf{u}}\otimes {\textbf{u}}\right)= & {} -\nabla p +\nu \nabla ^2{\textbf{u}}, \end{aligned}$$

19 $$\begin{aligned} \nabla \cdot \left( c{\textbf{u}} \right)= & {} \nabla \cdot \left( D\nabla c\right) . \end{aligned}$$

The quasi–static assumption allows the models to run with a large time-step controlled only by the velocity of the solid interface to save on computational time.

## Supplementary Information

Supplementary Information.

## Data Availability

The datasets generated during the current study are available in at Zenodo dataset archive (https://zenodo.org/record/6993528), the geometry creation scripts are on github (https://github.com/hannahmenke/Channeling2022) and an example input deck is on the GeoChemFoam wiki https://github.com/GeoChemFoam/GeoChemFoam/tree/main/Examples/.
