# Supplementary material for: Channeling is a distinct class of dissolution in complex porous media
Source: Sci Rep. 2023 Jul 13;13:11312. doi: 10.1038/s41598-023-37725-6 (PMC10344915; doi:10.1038/s41598-023-37725-6)
Supplement: Supplementary file 1 — Supplementary Information. [file 41598_2023_37725_MOESM1_ESM.pdf]

Appendix A: Model A and Model B Geometries

Geometry Creation

A uniform geometry was created with a uniform bead radius of 12 pixels placed on a diagonal grid with a spacing of 40 pixels and an offset of 20 pixels. A small random deviation of 2 pixels in the placement of the beads and 4 pixels in the radius of the beads was then introduced into this homogeneous model to allow for preferential flow paths to develop (Model A). It is interesting to note that this is the smallest random deviation that resulted in preferential flow paths, whereas with a lower deviation there was not enough heterogeneity for the wormhole or channeling regimes. Structural complexity was then increased by creating another model (Model B) using the same grid, spacing, and offset, but with random deviation of 6 pixels in bead radius and 12 pixels in bead placement. The model was set on a 1200 x 1200 pixel image which was then output at 10 times the resolution to preserve edges as a 12000 x 12000 pixel image. This image was then binned by 12 in each direction using ImageJ and padded by 2 on every side using Python to give the final model dimensions of 1004x1004 pixels. The resolution of the geometry was set to 3.5 μm per pixel, giving a domain size of 3cm$\times$3cm.

Each domain was meshed and the flow field calculated using the Open Source Computational Fluid Dynamics toolbox OpenFOAM [1] (Fig 2A & B). The distribution of pore throat sizes and velocities are presented in Fig 2C and the distribution of pore and grains sizes are presented in Fig 1. The scripts for creating the initial 12000x12000 geometries can be found on github. The original images with the radius, x, and y coordinates of each bead can be found on our Zenodo dataset archive.

Geometry Analysis with Image Analysis

The grains, pores, and pore throats were extracted from each time step in the simulations using a watershed segmentation algorithm and the Euclidean distance map of the grain and pore spaces was used to identify individual

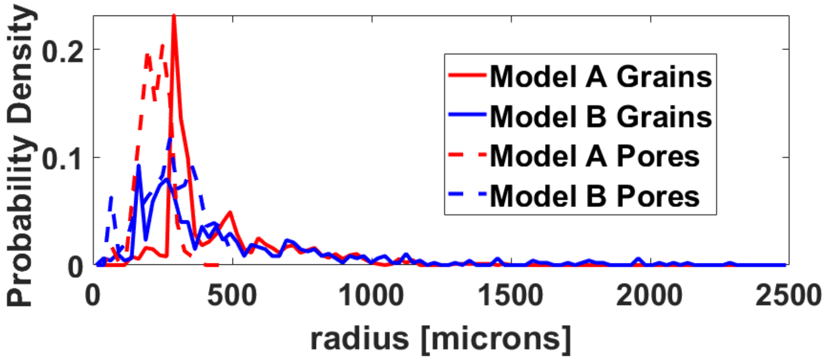

Fig. 1 The pore and grain radius distributions for the initial geometries of Model A and B.

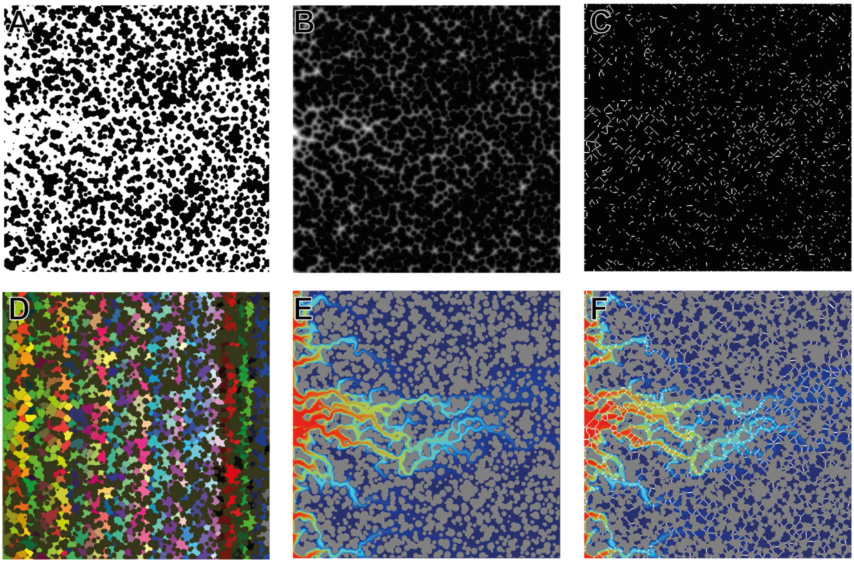

Fig. 2 (A) The pore space with pores in white and grains in black. (B) A Euclidean distance map was calculated on the pore space. (C) The local maxima of the distance map are designated the center of each pore. The boundaries between pores are designated as throats. (D) Each pore and throat is then individually identified, and local statistics calculated. (E) The concentration map (colored) is then overlain on the pore space with the grains in grey and (F) the local concentration statistics for each pore and throat are then calculated.

grains and pores with the boundaries between pores as throats. An example of this method with each initial geometry is shown in Fig 2. The statistics of the grain, pore, and pore throat size distributions along with the characteristic length and velocities (at Pe=1) are given in Table 1.

Geometry Analysis with Autocorrelation

Here we compute the autocorrelation of the grains and velocities for both Model A and Model B (Fig 3). Both models have an autocorrelation function that steeply decreases towards zero with lag, over a length scale equal to the grain spacing. Model A is statistically anisotropic, with an autocorrelation function with square symmetry and prominent sidelobes reflecting the underlying grid. Model B is statistically isotropic, with no sidelobes.

The autocorrelation function of the along-flow component of the velocity field is statistically anisotropic, with rectangular symmetry. The scale length in the along-flow direction typically is similar to the grain spacing but is larger (by a factor of about five) in the cross-flow direction, as is expected for channels. For Model A, the autocorrelation has sidelobes reflecting the underlying periodicity of the medium, with wavelength equal to the grain spacing. The autocorrelation for Model B is similar, but without the sidelobes.

Table 1 Table of initial geometry statistics

Statistic	Model A	Model B
Characteristic Length L [m]	1.125 x 10^{-4}	1.251 x x 10^{-4}
Pore radius mean [pixels]	6.4	8.3
Pore radius standard deviation	1.4	3.2
Pore radius skewness	-0.23	0.19
Pore radius kurtosis	3.5	2.9
Grain radius mean [pixels]	12.9	14.3
Grain radius standard deviation	6.3	12.6
Grain radius skewness	1.5	2.6
Grain radius kurtosis	5.4	12.4
Pore throat radius mean [pixels]	2.5	3.8
Pore throat radius standard deviation	1.2	2.4
Pore throat radius skewness	1.0	1.1
Pore throat radius kurtosis	4.7	5.0
Pore velocity U mean [m/s]	8.9 x 10^{-6}	8.0 x x 10^{-6}
Pore velocity U standard deviation	0.83	1.0
Pore velocity U skewness	2.6	3.5
Pore velocity U kurtosis	12	20

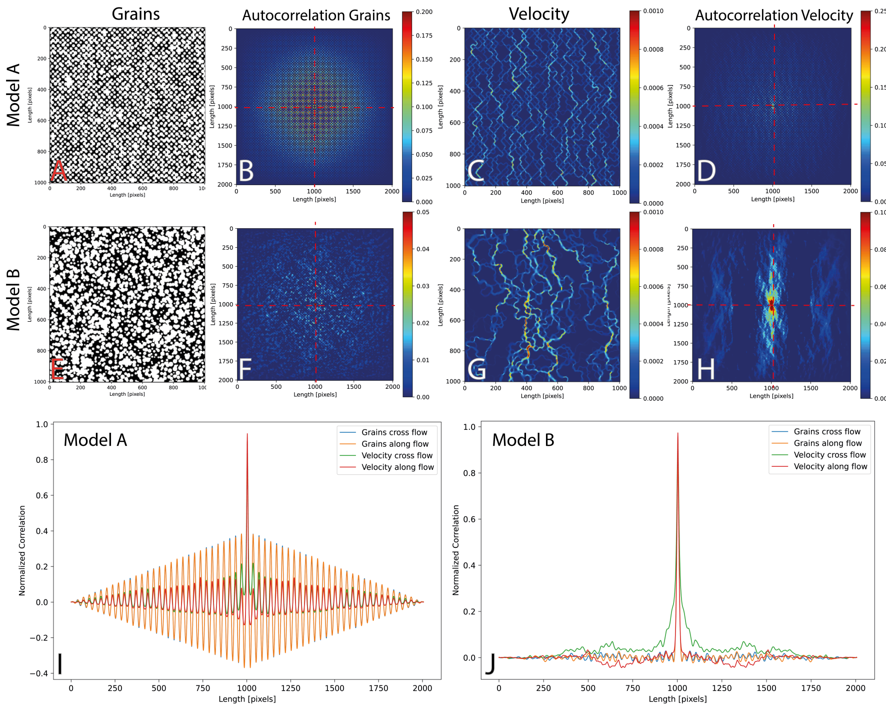

Fig. 3 The autocorrelation function for Model A and Model B shown for grains and velocity at Pe=1. A and E are the grains in white with the pores in black. B and F are the autocorrelation functions of the grains. C and G are the velocities in the direction of flow, D and H are the autocorrelation functions of the velocity. I and J are the autocorrelations of the grains and velocities for Model A and B respectively in each direction plotted from the centre points of the autocorrelations marked by red dotted lines on B, F, D, and H.

Velocity Distributions

Here we show the velocity rendering of each simulation at porosity=0.57 for Model A and Model B (Fig 5) and the corresponding PDFs of velocity(Fig.4). In both models we see a clear difference between the channeling and wormhole regimes, with the wormhole regime showing more of a fat tail as velocities not inside the wormhole are proportionally slower, while in the channeling regime the PDF has a higher more distinct peak more similar to the uniform regime as is expected with more preferential flow pathways throughout the model. As structural complexity increases the distribution widens for all regimes, as is expected for more diverse pore throat size distributions in more complex models.

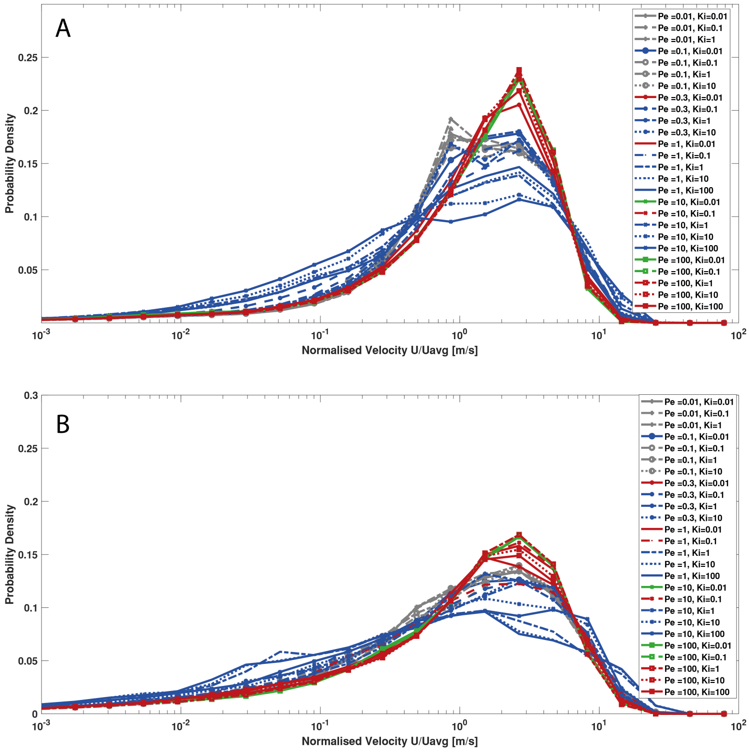

Fig. 4 The pdfs of velocity for (A) Model A and (B) Model B at Pe and Ki ranging from 0.01 to 100 at a porosity of 0.57. Simulations categorised in the compact, wormhole, and uniform regimes are shown in gray, blue, and green, respectively, while simulations that do not fit into any traditional regime are shown in red and designated channeling.

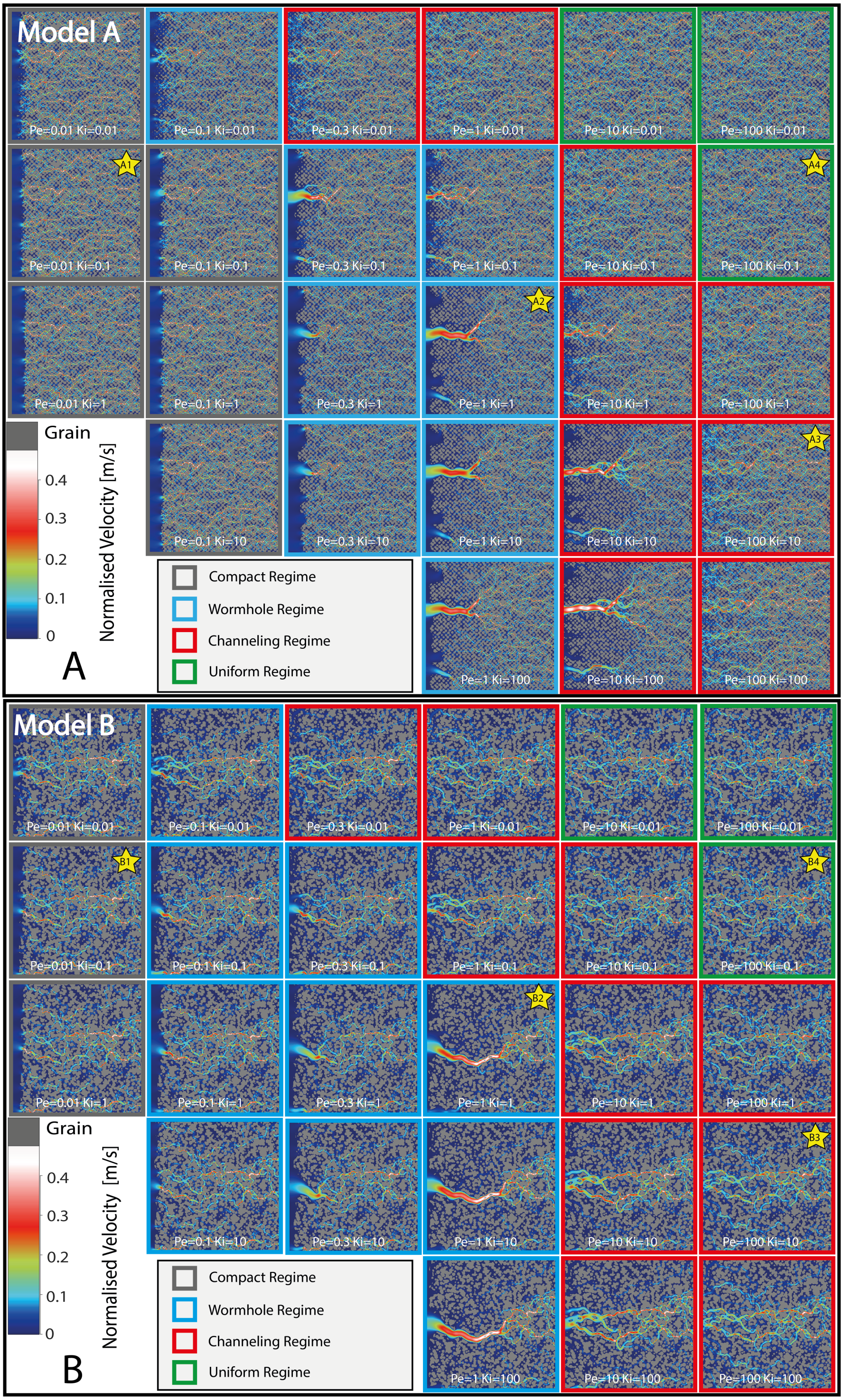

Fig. 5 Normalised Velocity during mineral dissolution in (A) Model A and (B) Model B at Pe and Ki ranging from 0.01 to 100 at a porosity of 0.57. The solid phase is rendered in grey and the velocity in colors. Simulations categorised in the compact, wormhole, and uniform regimes are outlined in gray, blue, and green, respectively, while simulations that do not fit into any traditional regime are outlined in red and designated channeling.

Appendix B: Numerical method

Meshing

The equations are solved using finite volume discretization over an unstructured hybrid mesh. To build the mesh, the solid surface is described using an *stl* image. First, a Cartesian mesh of resolution h is generated. The mesh is then snapped onto the solid surface using the *snappyHexMesh* utility [1], i.e. cell containing solid are then removed and replaced by hexahedral or tetrahedral cells that match the solid boundaries. An additional layer of cells of the same resolution h is then added around the solid boundary to improve the representation of the solid surface. To decide the resolution used for the initial mesh, a convergence study on porosity and permeability was conducted for Model B (Table 2). We observe that a resolution of 3 μm offers a good compromise between accuracy and size of computational mesh. Fig. 6 shows Model B with a zoom into a pore to observe the mesh at resolution 3 μm.

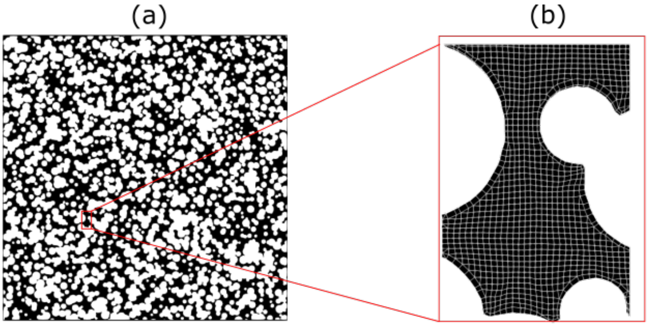

Fig. 6 Example of pore-space meshing (a) Full domain for model B (b) zoom and visualization of mesh inside a pore.

Table 2 Mesh convergence (Model B)

Resolution (μm)	Porosity	Permeabilty (m^2)	number of cells
6	0.432	3.17×10^{-10}	139k
3	0.455	5.64×10^{-10}	526k
2	0.457	5.68×10^{-10}	1141k

Arbitrary Lagrangian Eulerian method

The equations are solved using the Arbitrary Lagrangian Eulerian (ALE) method [2], implemented in GeoChemFoam (www.github.com/geochemfoam) and the full solution procedure is presented in Fig. 8. For each time-step, the

mesh points are moved with velocity $\mathbf{w}$, which satisfies the Laplace equations with boundary condition (Equ. (**??**))

$$\nabla \cdot D_m \nabla w_j = 0 \quad \text{j=x,y,z} \tag{1}$$

$$w_j = \mathbf{w}_s \cdot \mathbf{e}_j \quad \text{at } \Gamma, \tag{2}$$

where D_m is the diffusivity of the mesh motion, w_j is the j-directional component and $\mathbf{e}_j$ is the j-directional standard basis vector. With these equations, the mesh points will track the fluid-solid interface, and the mesh motion is diffused to avoid large volume ratio between neighbor cells. However, as the mesh points are displaced, the skewness of the mesh can increase and lead to failure of the transport solver. To avoid this, the mesh's skewness is checked at the end of each time-step, and the domain is fully remeshed upon failure. After remeshing, the velocity, pressure and concentration fields are mapped to the new mesh. In addition, topological errors can appear when two faces of the same mineral grain overlap, leading to failure of the flow or transport solver. To avoid this, the faces which are fully located in a topological error are eliminated before remeshing. These collapsing faces are identified by the following condition: a face defined as faceI collapsed if a ray leading from its center following its normal vector pointing toward the solid phase meets another face defined as faceJ at a distance lower than the grid size, and faceI and faceJ do not intersect. Following this remeshing algorithm, our numerical simulations are stable and topological errors are eliminated.

Time-stepping strategy

The simulations are performed using an adaptive time-stepping strategy based on the mesh Courant-Friedrich-Lewy (CFL) number defined as

$$mCFL = \frac{\mathbf{w}\Delta t}{h}, \tag{3}$$

where Δt is the time-step and h is the mesh resolution. The simulation are performed using a maximum $mCFL$ number of 0.005, which offers a good compromise between accuracy, robustness and efficiency. Fig. 7 shows a comparison of permeability evolution as a function of porosity for Model B at $Pe = 1$, $Ki = 1$ between mCFL=0.005 and mCFL=0.0025.

Appendix C: Robustness of ϕ, K and L for stochastically generated micromodel

The study presented in the paper is limited to one instance of each of two stochastic models (Model A and B). Future work will focus on extending the findings to any generated geometry and in particular on linking the dissolution regimes to the parameters of the stochastic distribution. For this, it would be essential that the geometrical parameters that are used in the calculation of

Pe and Ki, i.e. the porosity ϕ, and the pore-scale length L, vary over a range much less than an order of magnitude, so that the calculation of Pe and ki are robust over different instance of the same stochastic distribution. Table 3 shows the variation of porosity and pore-scale length for 12 instances of each stochastic distribution (Model A and B). For model A, ϕ varies between 0.430 and 0.445 and L varies between 1.04 and 1.15 $\times 10^{-4}$ m; for model B, ϕ varies between 0.451 and 0.473 and L varies between 1.14 and 1.41 $\times 10^{-4}$ m. This shows that the calculation of Pe and Ki will be robust, as ϕ and L varies on a scale much smaller than an order of magnitude.

Table 3 Porosity and L for 12 realizations of Model A and B)

Instance	Model A		Model B	
	ϕ	L ($\times 10^{-4}$m)	ϕ	L ($\times 10^{-4}$m)
1	0.437	1.11	0.455	1.22
2	0.439	1.11	0.473	1.41
3	0.436	1.11	0.455	1.34
4	0.432	1.10	0.457	1.29
5	0.445	1.13	0.466	1.28
6	0.440	1.11	0.465	1.23
7	0.436	1.11	0.464	1.31
8	0.437	1.12	0.468	1.34
9	0.439	1.15	0.460	1.31
10	0.436	1.10	0.451	1.14
11	0.430	1.04	0.463	1.25
12	0.438	1.08	0.472	1.22

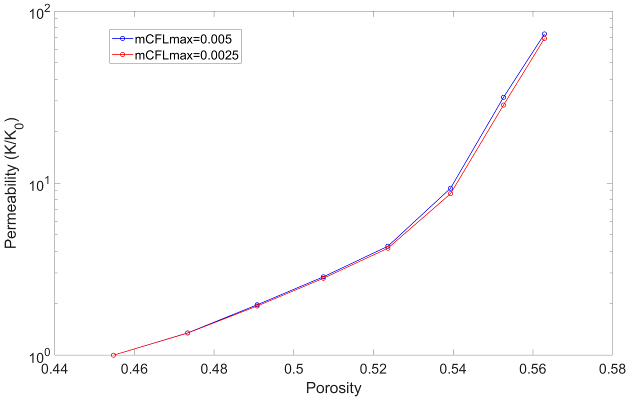

Fig. 7 Comparison of permeability evolution as a function of porosity for Model B at $Pe = 1$, $Ki = 1$ for two different maximum mCFL numbers.

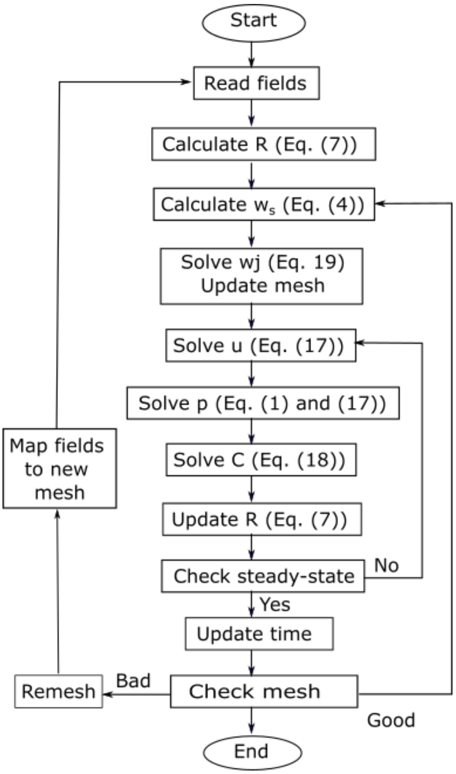

Fig. 8 Solution procedure for solving quasi-steady state dissolution using the ALE method.

Appendix D: Time Sequence Videos of Dissolution

Movies S1-8 show the dissolution time series for select simulations A1-A4 and B1-B4.

Movie S1: Visualisation of Model A Pe=0.01 Ki=0.1 evolution of porosity and concentration. The grains are gray, with the concentration field in color. The pore throats are extracted by a watershed algorithm on the Euclidean distance map of the pore space and superimposed in white. This is an example of the compact dissolution regime.

Movie S2: Visualisation of Model A Pe=1 Ki=1 evolution of porosity and concentration. The grains are gray, with the concentration field in color. The pore throats are extracted by a watershed algorithm on the Euclidean distance map of the pore space and superimposed in white. This is an example of the wormhole formation dissolution regime.

Movie S3: Visualisation of Model A Pe=100 Ki=10 evolution of porosity and concentration. The grains are gray, with the concentration field in color. The pore throats are extracted by a watershed algorithm on the Euclidean

distance map of the pore space and superimposed in white. This is an example of the channeling dissolution regime.

Movie S4: Visualisation of Model A Pe=100 Ki=0.1 evolution of porosity and concentration. The grains are gray, with the concentration field in color. The pore throats are extracted by a watershed algorithm on the Euclidean distance map of the pore space and superimposed in white. This is an example of the uniform dissolution regime.

Movie S5: Visualisation of Model B Pe=0.01 Ki=0.1 evolution of porosity and concentration. The grains are gray, with the concentration field in color. The pore throats are extracted by a watershed algorithm on the Euclidean distance map of the pore space and superimposed in white. This is an example of the compact dissolution regime.

Movie S6: Visualisation of Model B Pe=1 Ki=1 evolution of porosity and concentration. The grains are gray, with the concentration field in color. The pore throats are extracted by a watershed algorithm on the Euclidean distance map of the pore space and superimposed in white. This is an example of the wormhole formation dissolution regime.

Movie S7: Visualisation of Model B Pe=100 Ki=10 evolution of porosity and concentration. The grains are gray, with the concentration field in color. The pore throats are extracted by a watershed algorithm on the Euclidean distance map of the pore space and superimposed in white. This is an example of the channeling dissolution regime.

Movie S8: Visualisation of Model B Pe=100 Ki=0.1 evolution of porosity and concentration. The grains are gray, with the concentration field in color. The pore throats are extracted by a watershed algorithm on the Euclidean distance map of the pore space and superimposed in white. This is an example of the uniform dissolution regime.

References

[1] OpenCFD: OpenFOAM, the Open Source Cfd Toolbox, User Guide. OpenCFD Ltd, (2016). OpenCFD Ltd

[2] Starchenko, V., Marra, C.J., C., L.A.J.: Three-dimensional simulations of fracture dissolution. Journal of Geophysical Research: Solid Earth **121**, 6421–6444 (2016)
